# Comparative analysis of electrocautery and scalpel incisions in inguinal hernia repair

**DOI:** 10.6026/973206300221536

**Published:** 2026-03-31

**Authors:** Lav Gupta, Akhilesh Ratnakar, Pushpendra Kumar Patel, Talha Saad, Ajit Singh Morey, Satyendra Mishra, Ravikant Arjariya

**Affiliations:** 1 Department of Surgery, Bundelkhand Medical College Sagar, Madhya Pradesh, India; 2 Department of General Surgery, Bundelkhand Government Medical College, Sagar, Madhya Pradesh, India; 3 Department of ENT, Bundelkhand Medical College Sagar, Madhya Pradesh, India; 4 Department of Respiratory Medicine, Bundelkhand Medical College Sagar, Madhya Pradesh, India; 5 Department of Physiology, Bundelkhand Government Medical College, Sagar, Madhya Pradesh, India

**Keywords:** Inguinal hernia repair, scalpel incision, electrocautery incision

## Abstract

There is uncertainty about which incision technique-electrocautery or scalpel-offers safer and more effective outcomes in inguinal hernia repair, particularly with respect to operative duration, bleeding, postoperative discomfort, and wound-related complications. Therefore, it is of interest to compare the individuals having inguinal hernia repair utilizing blade and electrocautery incisions. Two groups were formed with 64 individuals in each group; group I (allocated to the patients who underwent surgical blade incision) and group II (allocated to the patients who underwent electrocautery incision). Statistically significant disparity was found among both the groups in regard to the incision time and intra-operative blood loss with a P value of less than 0.0001. We did not find statistically noteworthy disparity among the groups in regard to the inpatient stay duration, pain after surgery, painkillers necessity and post- procedural complications with a P value of 0.06, 0.53, 0.33 and 0.45 respectively. Skin incision with electrocautery is a swift procedure with the least complications post-procedural juxtaposed to the incision with a scalpel blade. So, utilizing the electrocautery for creating skin incision is as safe as the utilization of scalpel blade in patients undergoing treatment of inguinal hernia.

## Background:

A protrusion of intra-abdominal tissue via a fascial defect in the lining of the abdomen is referred to as an abdominal wall hernia. About 75% of abdominal wall hernias are inguinal, whereas various kinds arise at weak spots in the abdominal wall fascia [1]. Inguinal hernia repair is one of the most frequent surgical procedures performed globally, with over 20 million patients each year. For males and women, the lifetime chance of developing an inguinal hernia is 27-43% and 3-6%, respectively. Despite all the advancements, 10-12% of patients experience persistent discomfort after initial inguinal hernia surgery and 11% of patients experience a recurrence. Cutting electrocautery is rapidly becoming more widely accepted as an alternative to scalpels for skin incisions. The highest standard for generating surgical wounds was to make an incision using a scalpel [2]. Steel blades have traditionally been employed for creating surgical skin incisions. Surgical skin incisions are traditionally made with a scalpel, which may obscure the surgical site and contribute to bleeding. On the other hand, electrocautery offers advantage including a bloodless operative area as well as shorter incision times. Since it rapidly establishes hemostasis and facilitates quicker dissection, it is usually used for dissection. Furthermore, compared to scalpels, electrocautery diminishes the risk of infections conveyed to nurses and surgeons when handling tools [3, 4]. Nowadays, one of the key instruments utilized through surgeons to make skin incisions during herniorrhaphy is diathermy, which is simple and has a relatively avascular plane, especially for young teenagers [5]. As we operate in inguinal hernia surgery, the successful outcome of diathermy for subcutaneous and muscle opening is clearly documented [6]. Surgeons'perspectives on wound healing and infection highlight some questionable safety issues with skin incisions, notwithstanding its benefits [7]. Because electrocautery may trigger postoperative discomfort, infection and tissue damage, as well as interfere with wound healing and raise the risk of hypertrophic scarring, care should be taken with the heat employed and the duration of the incision [8]. In terms of pain after surgery, wound complications, incision time, intra-operative blood loss, duration of hospital stay and analgesics overall, the study contrasts and analyses scalpel and electrocautery incisions. Therefore, it is of interest to compare the analysis of electrocautery and scalpel incisions in inguinal hernia repair

## Materials and Methods:

Research Design and Sample this is comparative prospective research which was done on 128 patients for the duration of 1.5 years from January 2023 till June 2024. All patients presenting with uncomplicated inguinal hernia, age more than 20 years, irrespective of sex and willing to participate in study were the inclusions. Complicated inguinal hernia like irreducible hernia, obstructed hernia, strangulated hernia, recurrent inguinal hernia and Patients with co-morbidities like immunosuppressed, diabetes mellitus and on steroids and anticancer therapy were the exclusions. Sample size was estimated using the formula N = 4pq/r^2^is the formula, where q = (1-p) and r = precision (which was taken 5) (see PDF).

Prevalence rate was assumed 20% [9]. The value of N comes out to be 60.8 (about 61). We pursued our study on 64 subjects in each group. In all, 128 participants were included in the study. The following two groups were formed as Group A: Adults patients who were treated with scalpel incision and Group B: Adult patients who were treated with electrocautery.

Patients were randomly assigned to both the groups. Various parameters were evaluated as following:

[1] Incision time

[2] Intraoperative blood loss

[3] Inpatient stay duration

[4] Pain after surgery

[5] Painkiller necessities post-operatively

[6] Post-procedural complications.

a) Surgical site infection

b) Hematoma

c) Seroma

d) Purulent collection

## Ethical considerations:

Every safety measure was implemented to ensure that research participants' personal information remained private and protected. The ideals of the Declaration of Helsinki guided our research. Every subject gave their informed consent. The study received ethical clearance from the relevant ethical committee. Data Collection The surgical incision in both groups was created via skin, subcutaneous tissue, deep fascia and aponeurosis. By employing a sterile flexible ruler (in cm), length and depth of the incision were created and the incision time was defined as the initiation of the skin incision till the intended operation site was reached with complete haemostasis. Blood loss due to incision is defined as the loss of blood that comes at the time of incision which is computed as the differences between the dry and wet weight of the swabs (1 mg = 1 ml). Pain was evaluated on VAS on the day of discharge, where score 0 denotes no pain, 1 denotes mild pain, 2 specifies moderate pain and 3 depicts severe pain. A painkiller after surgery was infused via the intravenous route utilizing combination drugs of diclofenac sodium and aracetamol for both groups at first 24 hour. The post-procedural complications were assessed for 7 days post operatively. A t test and chi square test was applied using SPSS software to analyse the data and computation of the results.

## Results:

Table 1 (see PDF) exhibited the statistically significant disparity among both the groups in regard to the incision time and intra- operative blood loss with a P value of less than 0.0001. We did not find statistically noteworthy disparity among the groups in regard to the inpatient stay duration, pain after surgery, painkiller necessities and post-procedural complications with a P value of 0.06, 0.53, 0.33 and 0.45 respectively. This states that electrocautery incision exhibits less incision time and intra-operative blood loss for inguinal hernia repair. This research outcome highlights that electrocautery incision is better than scalpel blade incision for the treatment of inguinal hernia surgery. Although we found clinically significant disparities among the groups in terms of all the parameters but statistically significant disparity was noted only for incision timings and loss of blood at the time of procedure.

## Discussion:

Since the introduction of electrocautery, innumerable complications that occurred during and after surgery in the early 20th century have been significantly reduced. Many surgeons still chose scalpel blade incisions over electrocautery incision due to the worries of the post-surgery scarring and wound infection rates regardless of its worldwide use [4, 10]. Electrocautery utilization has better benefits which comprises of bloodless operative field, superior hemostasis, lesser pain after surgery and diminished complications post-procedural so its utilization is a better recommendation for skin incision over the electrocautery incision. The patients were randomly assigned to either the scalpel group (Group A) or the electrocautery group (Group B). In this research, mean age of the subjects were 45.2 years, with no significant disparities between the groups (group A and B) in regard to age, gender or physical characteristics like weight, height and BMI. We found statistically significant difference in incisional time between the scalpel skin incisions with the electrocautery incision. In similar research by Chauhan and Charpot revealed significantly shorter incision durations and less blood loss with electrocautery [11]. The amount of blood loss during the surgical procedure, exhibits statistically noteworthy difference in amount of blood loss between the scalpel skin incisions (group A) with the diathermy incision (Group B). In research done by Prakash *et al.* documented significantly lower blood loss per unit wound area with electrocautery, with no significant disparities in pain after the surgical procedure or wound infection rates [12]. The pains after the surgical procedure (or post-procedural pain) were assessed by VAS, the results analysed with chi-square test which uncovered a non-significant disparity between the two groups. Clinically, 48.4% of patients of group A had score 0 and 60.9% of patients of group B had score 0. Although there was difference between group A and B clinically but could not achieve statistically significant results. Research done by Yadav *et al.* uncovered that statistically significant outcome between scalpel and electrocautery group with regard to pain after surgery [4]. The mean painkiller necessities for Group A were 1.67±0.47 and for electrocautery was 1.77±0.67. The parenteral painkiller necessities showed no significant differences although it was clinically significant in our present research. Additionally, there were no significant disparities in complications of wound post-procedural or inpatient stay duration among both the groups. Research done by Yadav *et al.* discovers results in accordance to our present research in regard of inpatient stay duration [4]. In a similar study done by Zarei *et al.* too discovered the study outcomes are in line with our current research in context of inpatient stay duration [13]. In our research, we uncovered clinically noteworthy difference in the occurrence of post-procedural complications of wound but could not establish statistically significant disparity among both the groups (group A and group B). In the research performed by Zarei *et al.* outcomes were not significant among the two groups in relation to post-procedural complications of the wound which is in accordance to our current research outcomes [13]. In the present research, we discovered that electrocautery incision is a better choice of option in the context of timings of incision, loss of blood during the surgical procedure over scalpel blade incision. Other parameters also exhibited clinical variance in the values of both the groups but the difference was not much to be statistically significant. More research is required to be performed with large sample size in the field of diathermy and scalpel incision.

## Conclusion:

Electrocautery incision gives a better option to scalpel blade incisions for the surgery of inguinal hernia whether elective or emergency procedure. Electrocautery incisions are linked with shorter incision times, reduced bleeding, lower pain levels, decreased painkillers necessities and better cosmetic results. These do not enhance the complications post-procedural or extending inpatient stays. These outcomes favour the utilization of electrocautery as a highly effective and patient-friendly technique in the surgery of inguinal hernia. Electrocautery inguinal skin incision provides better-quality scar to the subjects, it is painless and without loss of blood.
